# Letter from the Professor of Mechanical Dentistry, of the Ohio Col. of Den. Sur

**Published:** 1854-01

**Authors:** 


### Correspondence.

*Editor of the Dental Register:*

Dear Sir: The Professor of Mechanical Dentistry of the Ohio College of Dental Surgery, hereby acknowledges a present to the College, of corrundum wheels, from S. Wardle and Co., manufactured by themselves; and in justice to them would say, that in quality and form, they are equal to any in use.

We would also acknowledge his obligations to Dr. J. M. Brown, for a present of files, and for furnishing all articles needed for the use of the College and Dental Students, at greatly reduced prices.

Both of these firms are manufacturers and dealers in Dental instruments and material, and every article used by the profession, of the best quality, can be procured of them.

And from the known ability of these gentlemen, we can cheerfully recommend them to the Dental Profession.

